# Improvements in Psychological and Occupational Well-being Following a Brief Yoga-Based Program for Education Professionals

**DOI:** 10.1177/2164956119856856

**Published:** 2019-06-11

**Authors:** Natalie L Trent, Sara Borden, Mindy Miraglia, Edi Pasalis, Jeffery A Dusek, Sat Bir S Khalsa

**Affiliations:** 1 Department of Research, Kripalu Center for Yoga & Health, Stockbridge, Massachusetts; 2 Brigham and Women’s Hospital, Harvard Medical School, Boston, Massachusetts

**Keywords:** psychological, health, yoga, educators, mindfulness, resilience

## Abstract

**Objective:**

The purpose of this study was to examine changes in psychological and occupational well-being in education professionals who attended a yoga-based program.

**Methods:**

Education professionals who attended a 3-day yoga-based RISE (resilience, integration, self-awareness, engagement) program were recruited to participate. RISE was administered at the Kripalu Center for Yoga & Health. Measures of psychological and occupational well-being, and health-related behaviors were completed before (baseline), after (post), and 2 months after RISE (follow-up). Forty-four participants completed baseline and post and were included in the analysis. Of those, 33 participants also completed the follow-up. Paired samples *t* tests were used to compare scores between time points.

**Results:**

Compared to baseline, at post, participants showed improvements in perceived stress, mindfulness, empowerment, positive affect, negative affect, self-compassion, total work engagement, vigor, sleep quality (all *P* values < .001), resilience, satisfaction with life, as well as exhaustion and professional efficacy which are dimensions of burnout (all *P* values < .01). At the follow-up, significant improvements were maintained for mindfulness, empowerment, self-compassion, sleep quality (all *P* values < .001), resilience, vigor, and exhaustion (all *P* values < .01) and positive affect, satisfaction with life, and work engagement (all *P* values < .05).

**Conclusions:**

These findings suggest that the yoga-based RISE program improves psychological and occupational well-being in education professionals. In addition, participants reported that attending RISE was feasible, they could continue using RISE practices long-term, shared them with work colleagues, and reported that RISE positively impacted both their daily lives and workplace environment. With these promising results, additional controlled research is warranted.

## Introduction

Education professionals, including teachers and principals, are exposed to a considerable level of stress. In the United States, more than half of educators experience excessive stress several days per week,^1^ and nearly 40% of educators leave the profession within their first 5 years of teaching due to stress.^2^ Chronic workplace stress puts educators at risk for burnout^3,4^ and many other health problems, including increased risk for disease and mortality.^5^ It is therefore imperative that education professionals acquire stress-coping resources to protect themselves from these negative consequences.

Yoga is a mind–body practice comprised of many components, including physical postures and exercises, breathing exercises, relaxation techniques, meditation, and mindfulness practices. Systematic research reviews support the use of yoga for promoting psychological, occupational, and physical health.^6,7^ Furthermore, there is growing evidence of yoga’s effectiveness for improving psychological, occupational, and physical health in professionals, including stress, mood, fatigue, and tension in police officer trainees^8^; perceived stress and depression in teachers^9^; improved self-care, mindfulness, and burnout in nurses^10^; reduced work-related stress in mental health professionals^11^; improved anger, anxiety, and sleep quality in armed forces members^12^; depression, stress, mindfulness, and self-compassion in mental health-care professionals^13^; and stress, resilience, mindfulness, affect, empowerment, and self-compassion in frontline professionals from multiple sectors including education.^14^

Despite these promising findings, there have only been a few studies on yoga programs specifically for education professionals.^9,15–17^ A recent study of a 15-day residential yoga program for primary school teachers resulted in improvements in mental well-being and state anxiety compared to a no treatment control.^17^ However, there was no assessment of occupational health or constructs hypothesized to mediate improvements in educator stress or anxiety, such as resilience or mindfulness. Furthermore, a 15-day residential yoga program would likely not be feasible for many education organizations. To date, the effect of a brief residential yoga program for education professionals has yet to be investigated. Yoga programs developed by the Kripalu Center for Yoga & Health (Kripalu) have resulted in improved psychological and physical health in professionals including police officers,^8^ military personnel,^18^ health-care workers,^13^ and a heterogeneous group of frontline professionals from education, corrections, and social services sectors.^14^

## Method

### Participants

**Figure 1.**
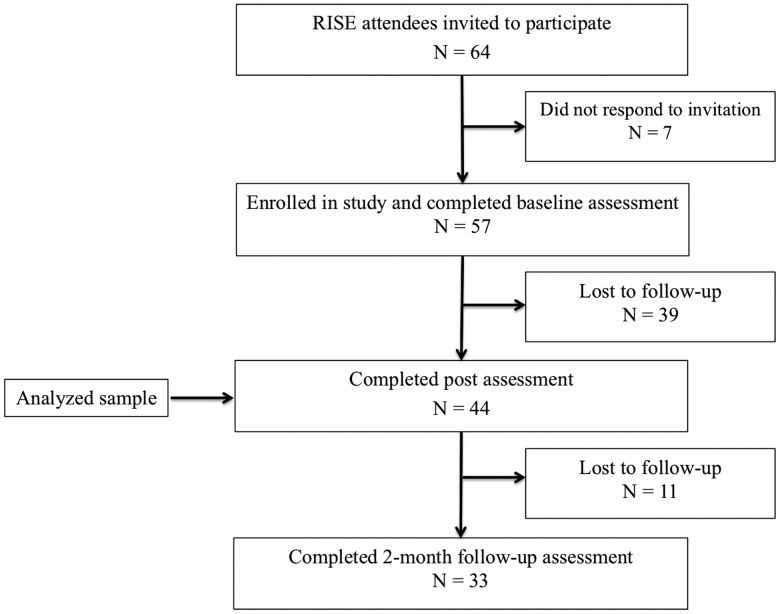
Participant CONSORT Diagram.RISE: Resilience, Integration, Self-awareness, Engagement.

Sixty-four professional educators (eg, teachers, principals) from nearby schools in the Pittsfield MA District School Board that attended a 3-day RISE program in 2017 and 2018 were recruited to participate in the study. Attendees were sent an e-mail containing information about the study 2 weeks before the start of the program and were sent a link to the survey 10 days before the start of the program. Of those recruited, 57 program attendees agreed to be in the research study and completed the baseline survey (89.1% of attendees). The final sample consisted of 44 participants (77.2% of participants enrolled) who completed the assessments at baseline and post. Of the 44 participants, 33 participants also completed the follow-up assessment (57.9% of participants recruited; see the CONSORT diagram in Figure 1). Participants were 95.5% female and an average age of 50.5 years (range 26–66 years). With respect to ethnicity and race, 97.7% were non-Hispanic, and 83.2% identified as White, 9.8% as Black, 2.4% as Asian, and 2.3% as multiracial. With respect to education, 88.3% had attended or completed graduate school, 7.0% completed a college degree, and 4.7% completed some college or an associated degree. With respect to occupation type, 45.5% were teachers, 31.8% were special education teachers of some kind, 15.9% were counselors, and 6.8% were administrators. The Institutional Review Board at Brigham and Women’s Hospital approved all aspects of this research.

### Intervention

The RISE program was delivered as a 3-day residential immersion program at Kripalu. Highly experienced, certified yoga teachers with at least 200-hour Yoga Alliance certification in addition to RISE facilitator training administered the RISE program. RISE attendees had the option of being housed at the retreat center in multiguest rooms or commuting from home. RISE included 5 hours of daily structured sessions of yoga postures, meditation, mindfulness practices, breathing techniques, and education about mindful communication, mindful sleep preparation, and mindful eating. The RISE program has also been described in detail elsewhere.^14^ The program time allocation was approximately 1/3 yoga instruction and/or practice, 1/3 didactic, and 1/3 experiential. Attendees were provided with all meals during the program and additional activities at the center (eg, hiking, dance, and cooking demonstrations) were available to participants when RISE was not in session.

### Data Collection

Surveys were administered online using the Partners HealthCare version of the software platform REDCap.^19^ Questionnaires were administered to participants at 3 time points: baseline (1–10 days preprogram), post (1–10 days following RISE), and follow-up (2-month post-RISE). The attendees were sent 3 reminder e-mails and 1 text message or phone call reminder if they did not respond to the survey invitation by e-mail.

### Measures

#### Demographics

Participants completed a demographic questionnaire, which included age, gender, race, ethnicity, and education.

### Psychological Health and Well-being

#### Stress

The 10-item version of the Perceived Stress Scale (PSS)^20^ was used to measure participants’ subjective level of stress. The PSS displays adequate levels of reliability and validity.^20^ For this study, we measured a Cronbach’s alpha of .87 for the PSS.

#### Resilience

The Resilience Scale (RS) was used to measure participants’ level of resilience, an ability that enhances individual adaptation to stress and challenging situations. The RS displays high levels of reliability and validity,^21^ and we measured a Cronbach’s alpha of .89 for the RS.

#### Positive/Negative affect

Positive and Negative Affect Schedule (PANAS) was used to measure positive and negative mood. The PANAS displays good internal consistency reliability and construct validity,^22^ with a Cronbach’s alpha of .86 for this study.

#### Mindfulness

The Five Facet Mindfulness Questionnaire (FFMQ) is a well-validated, reliable 39-item scale for the assessment of mindfulness.^23^ The FFMQ assesses 5 distinct facets of mindfulness: observing, describing, awareness, nonjudgment of experience, and nonreactivity to inner experience.^23^ The FFMQ shows good internal consistency reliability, with a Cronbach’s alpha of .92 for this study.

#### Empowerment

The Psychological Empowerment Scale (PES) is a 12-item scale that measures participants’ psychological empowerment, a sense of efficacy, competence, and self-determination. The PES displays acceptable levels of reliability and validity.^24,25^ We measured the internal consistency as Cronbach’s alpha of .84 for the PES.

#### Self-compassion

The Self-Compassion Scale–Short Form (SCS-SF)^26^ is a brief, well-validated version of the SCS. The SCS-SF includes 12 items that measure self-compassion. The internal consistency reliability of the SCS-SF is high, with a Cronbach’s alpha of .87.^26^ We measured a Cronbach’s alpha of .86 for the SCS in this study.

#### Satisfaction with life

The Satisfaction with Life Scale (SWLS) is a well-validated, short, 5-item scale that measures overall life satisfaction.^27^ A total satisfaction with life score is obtained by summing the item scores. We measured a Cronbach’s alpha of .89 for the SWLS.

### Occupational Health and Well-being

#### Burnout

The Maslach Burnout Inventory—General Survey (MBI-GS) is a well-validated and reliable 16-item measure of professional burnout.^4^ The MBI-GS measures burnout across 3 factors: exhaustion (EX), cynicism (CYN), and professional efficacy (PE). Items are rated on a scale from 0 (never) to 6 (every day). The MBI had a Cronbach’s alpha of .74 for this study.

#### Work engagement

The Utrecht Work Engagement Scale (UWES-9) is used to measure engagement in one’s occupation across 3 subscales: vigor (VI), dedication (DE), and absorption (AB).^27^ The UWES-9 displays good validity and reliability.^28^ We measured a Cronbach’s alpha of .89 for the UWES-9.

#### Job satisfaction

The single-item Job Satisfaction Scale^29–31^ measures job satisfaction with a single item, “At this moment, I am fairly satisfied with my job,” with the response rated from 1 (strongly agree) to 5 (strongly disagree). Lower scores indicate higher job satisfaction.

### Healthy Behaviors and Sleep Quality

The Lifestyle Questionnaire is a self-report questionnaire of health-related behaviors including physical activity, diet, and sleep disturbance that was developed by some of the authors of this study. The 4-item questionnaire assessed participants’ average minutes per day of physical activity, average daily vegetable and fruit intake, and sleep quality, rated from 1 (very good) to 4 (very bad), with lower numbers indicating better sleep quality.

### Program Impact and Continued Practice

An Impact Questionnaire assessed participants’ continued use of RISE skills and practices, sharing aspects of the program with others, and program feasibility. At post, on a visual analog scale from 0 (not at all) to 100 (very much so), participants indicated the degree to which they planned on practicing RISE skills and concepts (eg, breathing techniques, yoga classes), how likely they were to share what they learned in the program with others, and how feasible it was to accommodate the program into their schedule. At the 2-month follow-up, participants were asked to indicate the degree to which they had been practicing the RISE skills and concepts since the program, the degree to which they observed a positive shift in their workplace experience from being introduced to mind–body/mindfulness practices in the program, and the degree to which they shared what they learned in the program with others.

### Data Analysis

One-way analyses of variances (ANOVAs) were conducted to compare baseline scores between participants who completed measures at all 3 time points (full completers) and participants who only completed measures at the first 2 time points (partial completers). ANOVAs were conducted to compare baseline scores and change between participants who stayed at the retreat center and participants who commuted. Paired samples *t* tests were performed to compare participants’ scores between baseline and post and baseline and follow-up. Pearson correlations were performed between change scores (post—baseline, follow-up—baseline) for constructs of interest. The alpha level was set to .05 for statistical significance for all analyses. No adjustments were made for multiple outcome measures (to reduce type I error) based on the relatively small sample size and preliminary nature of this study.

## Results

### Participants’ Baseline Differences

A 1-way ANOVA was conducted to detect baseline differences between individuals who completed only the baseline and postintervention surveys (i.e., partial completers, n = 15) and participants who completed all surveys (i.e., full completers, n = 29). Partial completers showed higher levels of the absorption dimension of work engagement compared to full completers, *F*(1, 42) = 6.64, *P* = .014 (see Table 1). There were no other baseline differences between partial and full completers.

**Table 1.** Baseline Means and Standard Deviations of Partial Completers (n = 15) and Full Completers (n = 29), and Those Who Stayed as Guests (n = 29) and Those Who Commuted (n = 15).

Variable	Partial Completers	Full Completers	*P*	Guest	Commuter	*P*
	Mean (SD)	Mean (SD)		Mean (SD)	Mean (SD)	
PSS	15.93 (7.16)	15.86 (5.24)	.810	15.00 (6.23)	17.60 (4.85)	.167
RS	81.07 (9.22)	77.48 (11.71)	.545	80.72 (10.38)	74.80 (11.33)	.089
PA	35.33 (8.76)	34.03 (6.48)	.852	35.62 (6.81)	32.27 (7.82)	.148
NA	20.07 (7.02)	20.17 (6.04)	.912	19.38 (6.07)	21.60 (6.71)	.273
FFMQ	129.53 (17.04)	121.00 (15.79)	.133	125.34 (14.06)	121.13 (20.81)	.430
PES	5.35 (0.61)	5.36 (0.73)	.996	5.41 (0.63)	5.25 (0.79)	.471
SCS	37.33 (8.15)	38.48 (7.80)	.781	38.71 (6.87)	43.33 (9.02)	.121
SWLS	22.67 (5.56)	22.52 (7.05)	.223	20.07 (7.31)	23.86 (5.77)	.066
MBI-GS						
PE	29.93 (5.84)	28.14 (6.19)	.633	29.11 (6.43)	28.00 (5.46)	.575
EX	16.07 (8.66)	17.97 (6.63)	.749	16.54 (7.73)	18.87 (6.39)	.324
CYN	9.86 (6.00)	12.34 (8.90)	.460	10.39 (7.72)	13.67 (8.58)	.209
UWES-9	39.47 (9.14)	34.83 (7.69)	.091	38.24 (7.35)	32.87 (9.43)	.043
VI	11.20 (4.39)	10.00 (3.55)	.377	10.93 (3.91)	9.40 (3.64)	.215
AB	14.07 (2.71)	11.97 (2.49)	.032	13.55 (2.11)	11.00 (3.05)	.002
DE	14.20 (3.26)	12.86 (3.09)	.115	13.76 (2.82)	12.47 (3.72)	.204
Job satisfaction	2.29 (1.07)	2.75 (1.14)	.396	2.52 (1.09)	2.73 (1.22)	.561

Abbreviations: AB, absorption; CYN, cynicism; DE, dedication; EX, exhaustion; FFMQ, Five Facet Mindfulness Questionnaire; MBI-GS, Maslach Burnout Inventory—General Survey; NA, negative affect; PA, positive affect; PE, professional efficacy; PES, Psychological Empowerment Scale; PSS, Perceived Stress Scale; RS, Resilience Scale; SCS, Self-Compassion Scale; SWLS, Satisfaction with Life Scale; UWES-9, 9-item Utrecht Work Engagement Scale; VI, vigor.

Participants who stayed at the retreat center (i.e., guests, n = 29) reported higher levels of baseline work engagement than those who commuted to the Kripalu retreat center (i.e., commuters, n = 15) for the program, *F*(1, 42) = 4.35, *P* = .043 (see Table 1). There were no other significant differences between those who stayed at the retreat center and those who commuted for the program.

An ANOVA revealed no significant baseline differences between occupation type (teachers, special education teachers, counselors, and administrators); all *P* values > .20.

### Psychological and Occupational Health

**Figure 2.**
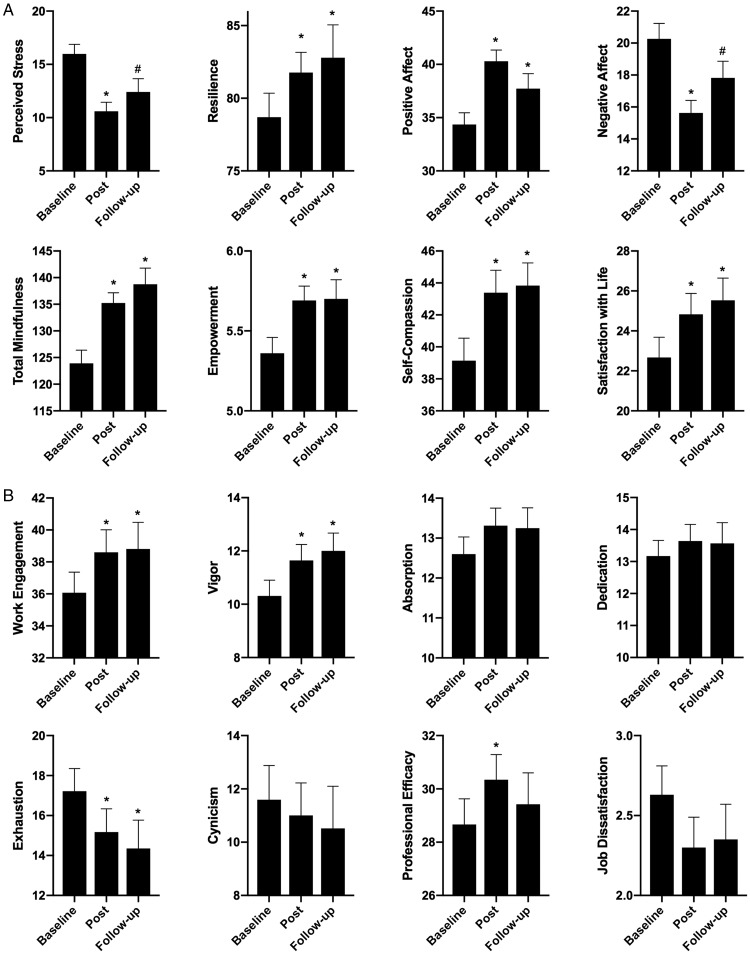
Means and Standard Errors of Psychological (A) and Occupational (B) Well-being Across Time Points (Baseline, Post, 2-Month Follow-up).

Means and standard deviations for the psychological and occupational health measures are displayed in Figure 2. With respect to the psychological well-being measures (Figure 2(A)), paired samples *t* tests revealed statistically significant improvements in perceived stress *t*(42) = −6.20, *P* < .001; resilience, *t*(43) = 2.85, *P* = .007; positive affect, *t*(42) = 5.53, *P* < .001; negative affect, *t*(42) = −5.36, *P* < .001; mindfulness, *t*(43) = 5.45, *P* < .001; empowerment, *t*(43) = 4.12, *P* < .001; self-compassion, *t*(41) = 4.77, *P* < .001; and satisfaction with life, *t*(41) = 2.98, *P* = .005, from baseline to post. With respect to the occupational well-being measures (Figure 2(B)), paired samples *t* tests revealed significant improvements in work engagement, *t*(41) = 3.40, *P* < .001, and its subscale vigor, *t*(41) = 3.77, *P* < .001; and the EX, *t*(40) = 2.95, *P* = .005, and PE dimensions of burnout, *t*(40) = 3.10, *P* = .004. There was no significant change in job satisfaction from baseline to post, *t*(39) = 1.57, *P* = .124.

At the 2-month follow-up, with respect to the psychological health measures (Figure 2(A)), significant improvements were maintained compared to baseline for resilience, *t*(27) = 2.92, *P* = .007; positive affect, *t*(32) = 2.41, *P* = .022; mindfulness, *t*(27) = 6.09, *P* < .001; empowerment, *t*(28) = 3.63, *P* < .001; self-compassion, *t*(31) = 3.96, *P* < .001; and satisfaction with life, *t*(31) = 2.15, *P* = .039. Levels of perceived stress, *t*(31) = −1.87, *P* = .07; and negative affect *t*(32) = −1.91, *P* = .06; were marginally significant (*P* values < .08) at the follow-up compared to baseline. With respect to the occupational health measures (Figure 2(B)), paired samples *t* tests revealed statistically significant improvements in work engagement, *t*(31) = 2.46, *P* = .020, and its subscale vigor, *t*(31) = 3.02, *P* = .005; and the EX dimension of burnout, *t*(30) = 3.15, *P* = .004. There was no significant change in job satisfaction from baseline to follow-up, *t*(30) = 1.12, *P* = .271. The correlations between change scores (post—baseline, follow-up—baseline) for questionnaires are displayed in Table 2.

**Table 2.** Pearson Correlations Between Participants’ Change Scores.

	PSS T2–T1	PSS T3–T1	RS T2–T1	RS T3–T1	PA T2–T1	PA T3–T1	NA T2–T1	NA T3–T1	FFMQ T2–T1	FFMQ T3–T1	PES T2–T1	PES T3–T1	SCS T2–T1	SCS T3–T1
PSS T2–T1	–													
PSS T3–T1	0.52**	–												
RS T2–T1	−0.19	−0.23	–											
RS T3–T1	−0.35	−0.54**	0.73*	–										
PA T2–T1	−0.62**	−0.53**	0.45**	.51**	–									
PA T3–T1	−0.40*	−0.63**	0.30	.51**	.74**	–								
NA T2–T1	0.58**	0.19	−0.07	−.08	−.37*	−0.17	–							
NA T3–T1	0.39*	0.74**	0.01	−.37	−0.28	−0.42*	0.45*	–						
FFMQ T2–T1	−0.23	−0.04	0.34*	.48*	0.30*	0.33	−0.11	−0.02	–					
FFMQ T3–T1	−0.24	−0.29	0.48**	.53**	0.53**	0.57**	−0.06	0.01	0.73**	–				
PES T2–T1	−0.05	−0.03	0.40**	.23	0.31*	0.31	−0.03	−0.01	0.34*	0.31	–			
PES T3–T1	−0.20	−0.46*	0.23	.26	0.27	0.37	−0.17	−0.14	0.28	0.29	0.71**	–		
SCS T2–T1	−0.52*	−0.50**	0.38*	.41*	0.46**	0.28	−0.38*	−0.01	0.65*	0.46*	0.34*	0.23	–	
SCS T3–T1	−0.39*	−0.56**	0.52**	.61**	0.61**	0.66**	−0.17	−0.33	0.57**	0.65**	0.36	0.25	0.56**	–

Abbreviations: FFMQ, Five Facet Mindfulness Questionnaire; NA, negative affect; PA, positive affect; PES, Psychological Empowerment Scale; PSS, Perceived Stress Scale; RS, Resilience Scale; SCS, Self-Compassion Scale; T1, baseline; T2, post-program; T3, 2-month follow-up.

*P < .05; **P < .01.

There were no significant differences in change scores between participants who stayed at the retreat center (guests) and those who commuted to the retreat center for the RISE program (commuters; see Table 3).

**Table 3.** Change Scores for Participants Who Resided at Kripalu (Guests, n = 29) and Participants Who Commuted to Kripalu (Commuters, n = 15).

Variable	Δ Post—Baseline		*P*	Δ Follow-up—Baseline		*P*
	Guest	Commuter		Guest	Commuter	
	Mean (SD)	Mean (SD)		Mean (SD	Mean (SD)	
PSS	−5.25 (6.22)	−5.60 (4.69)	.85	−1.24 (9.89)	−6.54 (6.67)	.13
RS	1.66 (6.59)	5.80 (7.57)	.07	3.12 (10.25)	8.64 (7.61)	.14
PA	5.00 (6.67)	7.73 (7.63)	.23	3.00 (7.80)	4.54 (7.29)	.60
NA	−4.57 (6.10)	−4.73 (4.95)	.93	−1.59 (6.43)	−4.19 (3.71)	.24
FFMQ	9.00 (10.53)	15.80 (18.10)	.12	14.71 (15.25)	21.91 (14.84)	.23
PES	0.29 (0.48)	0.42 (0.65)	.44	0.32 (0.52)	0.37 (0.51)	.79
SCS	5.30 (7.11)	6.07 (8.58)	.76	3.71 (8.10)	7.19 (5.06)	.22
SWLS	1.37 (4.91)	3.60 (4.12)	.14	1.53 (5.92)	4.19 (5.55)	.25
MBI-GS						
PE	1.23 (3.25)	2.47 (3.82)	.28	0.59 (6.33)	2.27 (4.84)	.46
EX	−1.92 (4.95)	−2.27 (3.58)	.82	−4.29 (6.47)	−3.00 (5.18)	.58
CYN	−0.62 (3.23)	−0.53 (7.27)	.96	−1.00 (4.76)	−2.00 (6.15)	.63
UWES-9 total	2.19 (4.95)	3.13 (4.64)	.55	4.83 (7.19)	3.18 (10.13)	.62
Vigor	1.22 (2.58)	1.53 (1.73)	.68	2.29 (3.67)	1.64 (3.72)	.65
Absorption	0.48 (2.39)	1.13 (2.45)	.41	1.71 (2.73)	0.91 (4.04)	.54
Dedication	0.48 (2.23)	0.47 (1.46)	.98	0.82 (2.48)	0.64 (3.11)	.86
Job dissatisfaction	−0.28 (1.57)	−0.28 (1.57)	.78	−0.56 (1.71)	−0.18 (1.17)	.53

Abbreviations: CYN, cynicism; EX, exhaustion; FFMQ, Five Facet Mindfulness Questionnaire; MBI-GS, Maslach Burnout Inventory—General Survey; NA, negative affect; PA, positive affect; PE, professional exhaustion; PES, Psychological Empowerment Scale; PSS, Perceived Stress Scale; RS, Resilience Scale; SCS, Self-Compassion Scale; SWLS, Satisfaction with Life Scale; UWES-9, 9-item Utrecht Work Engagement Scale.

### Healthy Behaviors and Sleep Quality

Paired sample *t* tests revealed no significant differences in average daily duration of exercise from baseline to post, *t*(41) = 1.84, *P* = .073; fruit intake, *t*(41) = 1.65, *P* = .107; or vegetable intake, *t*(41) = 1.77, *P* = .083. At follow-up, there were also no significant differences in average daily duration of exercise, *t*(30) = .59, *P* = .560; fruit intake, *t*(30) = .39, *P* = .702; or vegetable intake, *t*(29) = 1.15, *P* = .258, compared to baseline.

Paired samples *t* tests revealed a significant increase in sleep quality from baseline to post, *t*(41) = −4.37, *P* < .001. At the follow-up, participants’ increase in sleep quality remained significantly higher than baseline, *t*(30) = 3.72, *P* < .001 (see Table 4).

**Table 4.** Mean and SD of Exercise, Diet, and Sleep Quality Over Previous Week at Baseline, Post, and Follow-up.

Variable	Baseline	Post	Follow-up	P_1_	P_2_
	Mean (SD)	Mean (SD)	Mean (SD)		
Minutes of daily exercise	63.6 (75.08)	86.8 (95.94)	70.7 (97.38)	.073	.560
Daily servings of fruit	1.9 (1.04)	2.1 (1.01)	2.0 (1.02)	.107	.702
Daily servings of vegetables	2.6 (1.06)	2.8 (0.94)	2.9 (0.90)	.083	.258
Sleep quality^a^	2.3 (0.64)	1.8 (0.70)	1.8 (0.48)	.001	.001

Abbreviations: P_1_, *P* value between baseline and post; P_2_, *P* value between baseline and follow-up; SD, standard deviation.

^a^Lower values indicate higher sleep quality.

### Program Impact and Continued Practice

At postintervention, participants’ mean score for their overall preparedness to practice across all skills, practices, and concepts was 74.63, SD = 15.27. Participants were likely to share what they learned in the program with others, M = 89.72, SD = 12.96, and reported that it was feasible to accommodate the RISE program into their schedule, M = 90.07, SD = 12.08. At the follow-up, participants reported their overall practice across all skills, practices, and concepts as M = 69.49, SD = 16.18. They observed a positive shift in their workplace experience as a result of being introduced to mind–body/mindfulness practices in the program, M = 75.86, SD = 13.51, and they shared what they learned in the program with others, M = 70.06, SD = 23.24.

## Discussion

The main purpose of this study was to evaluate the effect of the 3-day RISE program on psychological and occupational health and healthy behaviors in educators. From baseline to post-RISE, participants showed improvements in stress, resilience, positive and negative affect, mindfulness, empowerment, self-compassion, satisfaction with life, PE and EX dimensions of burnout, work engagement, and sleep quality. From baseline to the 2-month follow-up, participants showed sustained improvements in resilience, positive affect, mindfulness, empowerment, self-compassion, satisfaction with life, EX, work engagement, and sleep quality. There were marginally significant improvements in stress and positive affect. Participants reported that attending RISE was very feasible, and that they continued to use many of the RISE practices regularly, shared them with others, and noticed that RISE positively impacted their daily lives and workplace environment.

Chronic stress is an increasing problem for education professionals and can result in health problems,^6,31^ as well as loss of productivity, and increased absenteeism.^32,33^ In the case of educators, workplace stress and burnout can also negatively impact their students, leading to psychological health issues and poorer academic performance.^34,35^ The results from this study provide further support for the recognized benefit of yoga for stress and burnout reduction in professional populations.^13,14,36,37^ Although the research is sparse, previous studies of yoga programs for educators have reported increased psychological and occupational well-being.^9,15–17^ While previous yoga and mindfulness-based intervention with educators showed similar results,^17,38–40^ they were administered across 3 to 20 weeks, whereas the current program was administered over only 3 days, having a comparable impact over a shorter period of time.

An aim of the RISE program is to cultivate skills and qualities such as resilience and mindfulness in professionals who improve their overall health and well-being both on the job and in their day-to-day lives. Resilience acts as a protective factor against the detrimental effects of stress,^41^ including the development of psychiatric disorders.^42^ Practical, brief, evidence-based programs that demonstrably increase employee resilience would benefit educators and their organizations. In this study, participants developed a greater level of resilience after attending the RISE program which was maintained 2 months after the program. In support of our findings, previous studies reported improved resilience following a workplace yoga intervention for university employees^36^ and a residential yoga program for frontline professionals including educators.^14^ Mindfulness is a key component of RISE that can be integrated into all daily activities such as when eating or communicating. Improvements in mindfulness following RISE were maintained at the 2-month follow-up. Results from previous studies have also demonstrated yoga’s ability to increase mindfulness across a wide range of populations, including educators.^14,43,44^

The benefits of yoga practice on psychological health are proposed to be mediated through increases in mindfulness, self-compassion, positive affect, and self-efficacy (i.e., empowerment).^45–47^ Although we cannot determine mediation from this study, increases in resilience were correlated with increases in all proposed mediators; mindfulness, self-compassion, positive affect, and empowerment. From baseline to post, decreases in perceived stress were correlated with increases in self-compassion, but not resilience, mindfulness, or empowerment. Improvements in self-compassion were correlated with improvements in most other health measures. From baseline to follow-up, decreases in perceived stress were correlated with increases in self-compassion, resilience, and empowerment, but not mindfulness; however, improvements in mindfulness were correlated with improvements in positive affect. The lack of significant correlations between perceived stress and mindfulness was unexpected, as previous studies report a relationship.^14^

Chronic workplace stress can lead to poor physical health, including the development of noncommunicable lifestyle diseases such as diabetes and cancer.^48^ In addition to reduction of stress, positive changes in lifestyle habits, including increased exercise, nutrition, and proper sleep can prevent negative health outcomes.^49^ We observed improvements in sleep quality immediately following and up to 2 months following RISE. A systematic review of the effect of MBIs on sleep quality reported improvements in sleep quality following yoga interventions.^50^ Unexpectedly, there were no changes in exercise or vegetable and fruit intake. Results from other yoga interventions have indicated that yoga increases physical activity and improves diet.^19,51^ It is unclear why we did not observe improvements in exercise and diet. However, the current intervention was delivered over 3 days with the option to commute, compared to the previously published study, which was delivered as a residential program over 5 days with all participants staying as guests.^14^ Therefore, participants may not have had as much time at the retreat center to shift their exercise and diet. Participants who commuted may not have stayed for meals at the retreat center, in which fresh fruits and vegetables were available, or may not have engaged in as much physical activity as the guests, such as hiking or using the exercise facility.

Given the preliminary nature of this study, there are several limitations that require discussion. First, the use of a single-arm design with a modest sample size is a significant limitation in not controlling for possible nonspecific effects of the intervention and limiting statistical power, although still useful for conducting a more informed future randomized controlled trial. This study provided necessary early stage feasibility data for future work, which will compare educators attending RISE to passive and active control groups, such as educators attending a retreat without RISE. Second, participants were a heterogeneous group of education professionals, categorized as teachers, special education teachers, counselors, and administrators. We observed no baseline differences across each category; however, a future study with a larger population with an equal number of participants from each category could enable a proper statistical analysis to detect differences. Another limitation is that participants did not complete practice logs of their use of the practices outside of the program, preventing an assessment of home practice on changes in outcome measures. However, the overall assessment of use across practices provides some indication at this early stage of research. Finally, the reliance on self-report measures to assess changes in psychological and occupational health is limiting, and future studies will aim to include objective measures such as physiological correlates of health, workplace absenteeism, productivity, student behavior, and academic performance. Future work may also implement a multitiered RISE program delivered to administrators, teachers, and students of the same school and will include an assessment of changes at the individual and institutional level.

Taken together, the results of this study indicate that the yoga-based RISE program improved psychological and occupational health and well-being, and sleep quality in education professionals immediately following and 2 months following the program. Furthermore, participants reported that the program was very feasible and that they continued to use the practices and techniques in their professional and personal lives. Workplace stress remains a growing concern for many organizations, often leading to a loss of productivity and absenteeism. Effective and feasible programs that build resilience in education professionals to reduce stress are needed to prevent burnout and detrimental outcomes to health and well-being. The results of this study support the use of Kripalu’s yoga-based RISE program for improving psychological health and well-being in education professionals.
